# Mr. Schaw, on Cold Affusion

**Published:** 1800-07

**Authors:** James Schaw

**Affiliations:** Surgeon. His Majesty's Ship, Tourterelle, Spithead


### *To the Editors of the Medical and Phyſical Journal.*

Gentlemen,

If the following, in your opinion, can be of the ſmalleſt ſupport to the uſe of cold affuſion in fevers, and be deemed worthy a place in your truly valuable Journal, its inſertion will oblige,

Gentlemen,

Your very obedient ſervant,

JAMES SCHAW, Surgeon.

*His Majeſty's Ship, Tourterelle, Spithead, May* 12, 1800.

The affufion of cold water on the furface of the body in typhus and other fevers, fo ably treated of, and recommended, by Dr. Currie, appears to me a fubject deferving much attention, both as a fimply efficacious remedy, and for the eafe with which it may always be procured; it therefore becomes a duty incumbent on every practitioner who has experienced its effects, to communicate the refult of his practice to the public.

From various opportunities which I have had of obferving this method of treatment, and from having frequently put its effects to the teft myfelf, I feel fully warranted in afferting that, in every cafe where it is judicioufly employed, its efficacy will invariably be evinced. In country practice, when the general affufion could not be ufed on account of the prejudices of the lower order of men, I have feen different inftances, where even the partial application of cold water to the face, neck, breaft and arms was of infinite fervice, and, indeed, I hardly ever faw it ufed without fome very palpable advantage arifing.

In the Medical Journal for laft March, I obferve a paper from fome perfon, affuming the appellation of Viator, wherein the author feems to queftion the propriety of the practice, and fuggefts that it may be productive of obftinate diarrhoea when unfuccefsful. Without entering into controverfy with your anonymous correfpondent, I fhall only recommend to him to give it a fair trial, ere he ftarts any more objections, when, I may fafely prognofticate, he will find his theoretical infinuations quickly vanifh before the peremptory and uncontroulable evidence of fact and experience. A late traveller, who feems to differ a little in this opinion with our Viator, informs us, the practice was by no means an uncommon one in Abyffinia; and, if I miftake not, he fays, the water was copioufly thrown over the patient as he lay in bed, in all ftages of highly putrid and other fevers, with, I believe, manifeft advantage. Its good effects are very well known to many who vifit more fouthern climes. A meritorious practitioner of my acquaintance, who has made repeated voyages to Africa, affured me that he found the moft beneficial confequences refult from a copious ufe of it. He ufed to place the patient under a pump, and allow the water to play over him in a full ftream, or dafh it over his body in buckets full, with admirable effects.

Mr. Martineau, in a paper inferted in the Medical Journal for January laft, feems to approve its application in cafes of internal inflammation, and fays, that no inconvenience arofe from it even where pneumonic complaints were prefent. With all due deference to Mr. M's opinion, and without any intention to implicate in the leaft the accuracy of his obfervations,

I beg

I beg leave to remark, that, in three cafes which fell under my own infpection, where catarrhal fymptoms attended the fever, the cold ablution appeared to do evident mifchief, by increafing the cough, and confequent irritation, to fuch a pitch as almoft totally precluded reft; which, of neceffity, compelled the remedy to be abandoned.

The beneficial effects of cold lavation in febrile diforders, may, perhaps, be farther exemplified by an inftance that occurred in the Royal Infirmary at Edinburgh. The difeafe was fmall-pox; the fubject a Negro lad about 17. He was brought into the Infirmary with all the fymptoms of a pretty acute fever. No eruption was prefent, and the affufion of cold water was directed by, I believe, Dr. Home. This, to the beft of my recollection, was performed twice, when the eruption appeared, and put a ftop to its further ufe. The puftules were extremely few in number, and the attendant fever fo exceedingly mild, that the patient walked about the ward during the whole continuance of the difeafe.—But to relate a cafe:

A. M. a failor, ætat. 30, of a robuft healthy conftitution, was attacked, on the 22d of October laft, with all the ufual fymptoms of fever, which he attributed to cold. He had an emetic given, which operated very well as fuch, but produced no other fenfible effect. On the 23d, in the morning, his pulfe was 116 in the minute, his fkin very hot; he had great thirft, and complained of fevere head-ach; had a ftool during the night. My hand acted the part of a thermometer, and I directed the application of cold water, which was performed while I felt his arm with one hand, and held my watch in the other to mark the change I expected. The affufion was fcarce finifhed when his pulfe fell to 90; he was then dried and put to bed. I vifited him in half an hour, when his pulfe was 92, and regular, fkin cool, head-ach much relieved, and he felt wonderfully refrefhed. Six o'clock, P. M. pulfe 100, heat above natural, head-ach increafed fince the afternoon. The operation was repeated with the fame effect as in the morning, reducing the pulfe to 70. In an hour afterwards, when I again vifited, he faid he felt "very eafy," his head-ach was nearly removed, and the heat of his fkin was very little, if at all, above natural; I ordered an opiate to be given at bed-time.

24th. Eight o'clock, A. M. pulfe 76; flept well during the night, has little head-ach, fkin very little above natural heat; had a ftool this morning.

Rep. Affufio aquæ frigid.

7 P. M. Pulfe 72, fkin of natural heat, no head-ach; has been walking about great part of the afternoon, and fays he feels "quite ftrong." No farther application was neceffary.

D. L. ætat. 25, of a healthy and ſtrong conſtitution, was attacked, in the night of the 21ſt of February laſt, with cold ſhivering, followed by increaſed heat, head-ach, nauſea, and ſlight vomiting. I ſaw him about nine next morning. The vomiting had ceaſed; the nauſea was likewiſe gone; pulſe 112, and ſtrong; ſkin hot; face fluſhed; breathing a little hurried, but without pain of breaſt, or cough; head-ach very acute; tongue clean, of a bright red colour; much thirſt. I ordered, and ſaw performed copiouſly, the cold affuſion. The ſhock was conſiderable, but the effect almoſt inſtantaneous. The pulſe immediately fell to 80, and the head-ach was greatly relieved.

At one the pulſe was 90, and the heat above the natural ſtandard; I repeated the remedy; which ſucceeded ſo well, that in the evening the head-ach was removed, the ſkin of natural heat, and he recovered without farther application.

Theſe two caſes, I think, ſhew pretty pointedly the advantage of an early employment of this ſimple and uſeful remedy. I have made uſe of it too in the more advanced ſtages of typhus, and, I think, with very happy effects. It evidently mitigated the violence of the febrile ſymptoms, and appeared to ſuppreſs the tendency to delirium; and although the diſeaſe generally ran out its courſe, yet it ſeemed to aſſume a milder form, and to be attended with leſs danger, when this treatment was adopted.

I might have related other caſes of the ſame nature, but as the events in all were pretty ſimilar (allowing for a ſlight variation in circumſtance), and as I am fearful of exceeding the limits of a ſheet, I defer, till ſome future period, ſaying any farther on the ſubject.

Should this be honoured with your notice, I ſhall be ſtimulated to renew my efforts in the ſame cauſe, or any other which chance or opportunity may place within my reach.

The inaccuracy and want of connexion which may appear above, I doubt not will be liberally excuſed, when it is conſidered how ill calculated a nautical life is for application or ſtudy.

